# Short Peptides for Hydrolase Supramolecular Mimicry and Their Potential Applications

**DOI:** 10.3390/gels9090678

**Published:** 2023-08-23

**Authors:** Paola Alletto, Ana Maria Garcia, Silvia Marchesan

**Affiliations:** 1 Chemical and Pharmaceutical Sciences Department, University of Trieste, 34127 Trieste, Italy; 2 Instituto Regional de Investigación Científica Aplicada (IRICA), Universidad de Castilla-La Mancha, 13071 Ciudad Real, Spain; 3 Facultad de Ciencias y Tecnologías Químicas, Universidad de Castilla-La Mancha, 13071 Ciudad Real, Spain

**Keywords:** peptides, enzymes, mimicry, mimetic, self-assembly, supramolecular, chirality, D-amino acids, gels, catalysis

## Abstract

Hydrolases are enzymes that have found numerous applications in various industrial sectors spanning from pharmaceuticals to foodstuff and beverages, consumers’ products such as detergents and personal care, textiles, and even for biodiesel production and environmental bioremediation. Self-assembling and gelling short peptides have been designed for their mimicry so that their supramolecular organization leads to the creation of hydrophobic pockets for catalysis to occur. Catalytic gels of this kind can also find numerous industrial applications to address important global challenges of our time. This concise review focuses on the last 5 years of progress in this fast-paced, popular field of research with an eye towards the future.

## 1. Introduction

Hydrolases are important enzymes that are involved in degradative processes. They find numerous industrial applications. Some are well established, and others are being developed in academic labs worldwide, as discussed further below. These enzymes are clearly attractive for industrial production since they offer a green route to a variety of products, and they do not persist in the environment after use. A few hydrolases can be produced on a large scale at a relatively low cost [1,2], but many more are prohibitively expensive for large-scale applications [3]. Furthermore, enzymes present other limitations, too. They are often susceptible to degradation, and they typically require mild physicochemical conditions to maintain activity. However, examples of biocatalysts of this kind with enhanced stability do exist [4]. Moreover, proteins can also trigger immune response and allergic reactions, so their presence in commercial products has to be carefully evaluated, depending, for instance, on the type of consumer exposure, duration, and on the amount of enzyme present [5].

**Figure 1:**
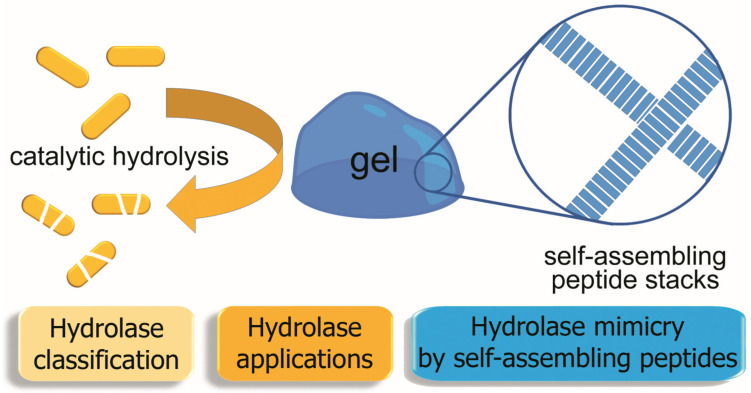
Outline of this concise review of hydrolase mimicry by self-assembled peptide hydrogels.

For all these reasons, the use of peptides as enzyme mimetics offers an attractive alternative, especially if they are very simple and consist of just a few amino acids. For instance, in the case of sequences as short as 2–3 residues, they can be easily produced by the economical liquid-phase synthesis that is amenable to large-scale production [6,7] or also by biotechnological means [8]. Such short peptides are unlikely to be immunogenic due to their reduced molecular size [9]. In particular, those capable of self-assembly into nanostructured yet macroscopic gels (Figure 1) have attracted great attention over the last two decades as protein mimics that form innovative and functional soft materials. They share many advantages with enzymes, such as biodegradability and environmental friendliness [10,11,12,13]. Furthermore, their functionalities and biodegradation rate can be fine-tuned with the inclusion of non-canonical amino acids in their sequence [14,15,16].

The idea of using short peptide bioactive motifs in place of proteins has attracted the interest of many researchers, and recent reviews are available in the literature [17,18,19,20]. In particular, in the case of enzymatic mimicry, it can be advantageous to exploit peptides’ supramolecular organization through self-assembly so as to recreate hydrophobic pockets where reagents are confined and protected from the bulk solvent for catalysis to occur [21]. Mastering this process is highly attractive given the possibility of positioning different functionalities designed ad hoc [22,23] thanks to the great molecular diversity of peptides and the easy and modular solid-phase synthesis. Finally, if enzymatic activity arises with supramolecular organization, in line of principle, it should be possible to design biocatalysts that can be switched on/off with assembly/disassembly cycles, and that can even work in tandem to mimic the natural modes of biochemical cascades [24]. However, challenges do exist, such as those related to the diffusion of reagents and products within the soft material for maximal biocatalytic efficiency [25].

This area of research has gained wide popularity, and numerous research works have been produced over the years. However, the field is rapidly evolving, and for this reason, in this concise review, we analyze in particular the last 5 years of progress in the field, with an eye on emerging and future applications.

## 2. Hydrolase Classification

**Figure 2:**
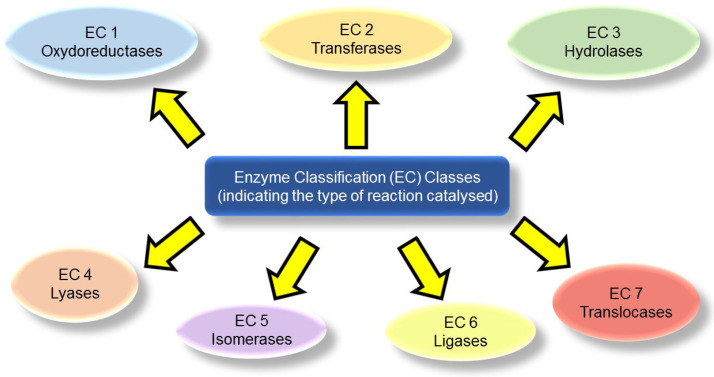
The Enzyme Commission (EC) number classifies enzymes into seven classes depending on the type of reaction they catalyze. Reproduced from [27].

Enzymes are numerous and form a highly diverse group of biocatalysts. Great efforts have been devoted to the development of international standards for their unambiguous classification and nomenclature. The most widely adopted system is the Enzyme Commission (EC) that can be easily explored on the ExplorEnz website [26] that was introduced by The International Union of Biochemistry and Molecular Biology (IUBMB). The EC system comprises four numbers that specify the catalyzed reaction (i.e., EC X.X.X.X). The first one generally defines the type of reaction. Hydrolases are identified by EC 3, which refers to hydrolytic reactions (pale green in Figure 2).

**Figure 3:**
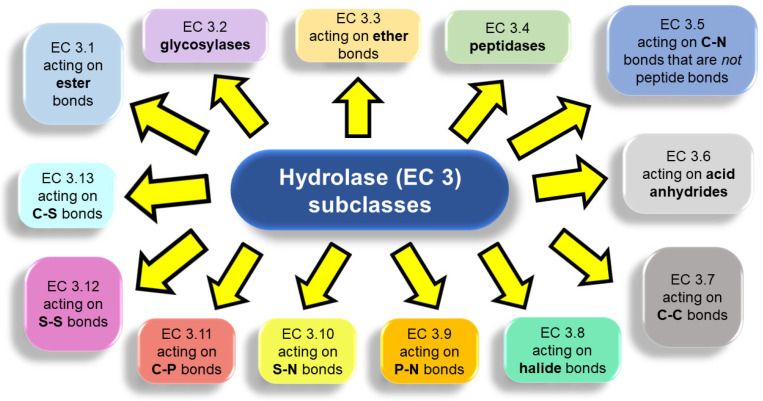
Hydrolases comprise thirteen subclasses depending on the type of chemical bond hydrolysis they catalyze.

The second number further specifies the subclass according to the type of functional group or molecule that takes part in the reaction (Figure 3). Hydrolases can be problematic because many of them present an extremely wide specificity [26].

Enzyme subclasses (EC X.X) are then divided into sub-subclasses through the third number of the EC system that further specifies the reaction that the enzyme catalyzes, for example, according to the chemical group to which it is transferred or the acceptor that receives it. Finally, the last component of the EC system can be considered as a serial number that identifies the specific enzyme [26].

It is evident how useful the EC system can be for quickly grasping the type of activity that an enzyme has from its EC number. However, its main limitation is that it is not always unambiguous. It is thus not surprising that EC classes [28] and sub-classes [29] are continuously being re-organized. Despite the tremendous importance of unambiguous enzyme classification, there is still some resistance within the biochemical community itself against recognizing it [30].

Another limitation is that the EC system does not include enzymes that catalyze non-natural reactions [30]. However, more and more of these are being reported, especially thanks to the advent of directed evolution [31]. Clearly, this area is expected to grow given the urgency to convert industrial processes towards greener routes.

Machine learning methods and other modern in silico tools have been used to predict and understand the function of enzymes [32,33,34]. Databases have been created to complement the information stored in ExplorEnz, such as *BRENDA* [35], *NIST* [36], *HUGO* [37], *NCBI* [38], *KEGG* [39], *MEROPS* [40], and *Expasy* [41].

## 3. Hydrolase Applications

Lipases are amongst the hydrolases that have found the widest application in several industrial sectors, as described further below. These enzymes can be produced by a variety of sources, with microbes and fungi often being preferred for several of their characteristics:

- Low-cost;
- Robust;
- Tolerant to organic solvents;
- Promiscuous towards the catalysis of a variety of reactions.

The latter ones include not only ester hydrolysis but also esterifications, amidations, transesterifications, acidolysis, alcoholysis, aminolysis, and others [42]. These enzymes have been widely studied and improved over the years through mutagenesis [43] and chemical modifications [44]. They have also been immobilized on a substrate for improved stability and for the ease of recycling [45,46]. In particular, the use of gel phases to immobilize lipases for enhanced performance has been reported, including organogels [47], emulsion gels [48], hydrogel microspheres [49,50], ionic gels [51], discontinuous hydrogel-organogel hybrids [52], and composite gels [53].

**Figure 4:**
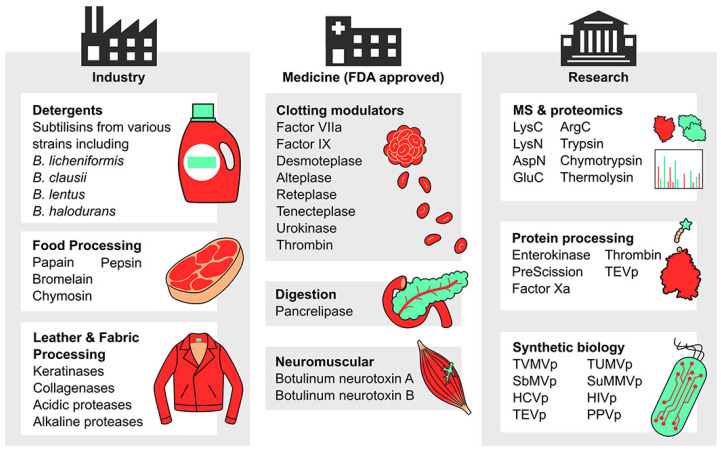
Proteases have been widely applied in several industrial sectors. Reproduced from [54].

A second class of enzymes that has been widely applied in diverse industrial sectors comprises proteases (Figure 4). The predominant position of proteases in the enzymes’ market is also the fruit of decades of protein engineering studies that enabled the production of mutants with altered substrate specificity and with improved stability to address industrial needs [54].

### 3.1. Chemical Synthesis

Lipases are cheap and sturdy biocatalysts that withstand a wide range of physicochemical conditions, including organic solvents, pH, and temperature differences. Thanks to their ability to convert one enantiomer at a significantly faster rate than the other, they are very convenient in the production of chiral compounds and in the resolution of racemic mixtures. They have thus found industrial use for the production of single molecules containing alcohol groups, such as fragrances (e.g., isopulegol) and bioactive compounds (e.g., retinol), but also compounds with acids, esters, and lactones, ranging from drugs to herbicides and polymers [55]. Readers interested in the industrial use of lipases to produce pharmaceuticals and their building blocks can find details elsewhere [56,57].

Proteases are also emerging as convenient and eco-friendly tools for the preparation of bioactive compounds, but, in contrast with lipases, they are studied especially in synthetic biology and biotechnology for the production of biotherapeutics [54].

### 3.2. Medicinal Uses

Proteases are the hydrolases that have found larger use as biotherapeutics, as shown in Figure 4 [54]. The main uses are as clotting modulators, digestive aids, and wound debridement [58,59]. In particular, the use of gel formulations has been studied for topical applications and for wound treatment [60,61,62].

Lipases have been proposed to treat hair loss and diseases of the scalp skin. They are contained in topical creams to locally reduce deposits of fat. They have been used as digestive aids and as adjuvants for the treatment of cancer of the digestive system, such as pancreatic cancer. Lipases can be used as biomarkers, too, since they are present in higher levels in the case of ongoing pathologies affecting the pancreas or the skin, where the increased hydrolytic activity leads to higher local acidity and inflammation [55].

### 3.3. Food Industry

Lipases are widely used by the dairy industry, for instance, to enhance the flavor of cheese, for the hydrolysis of milk fat, and to modify the fatty acid chain content. The oil and fat industries make large use of lipases, too, usually to improve their products quality and to modify their chemical content as needed through reactions of hydrolysis, esterification, or transesterification. In particular, lipases are extensively applied to produce cheap analogs of the expensive cocoa butter or human milk fat from vegetable sources. Another important application is the enhancement of food products based on eggs, where key aspects such as gelation, foaming, and emulsification can be controlled by the lipid content. Finally, lipases are applied also to reduce the fat content in dietary products, fish and meat, and to enhance the flavor of processed meat [55].

Proteases are another hydrolase class that has been long used in the food industry, for instance, to digest casein in milk processing to produce cheese but also in baked products to hydrolyze gluten and, in general, in a variety of foodstuffs to obtain easier digestibility, with hydrolyzed milk for infants being a representative example [63].

### 3.4. Beverage Industry

Several hydrolytic enzymes find application in the production of beverages, especially those that are produced from fruits, both fermented and not. Proteases, pectinases, amylases, cellulases, and hemicellulases can be used for various purposes. They include reduction of viscosity for easier processing, clarification, and improvement of the organoleptic and nutritional quality of the final product [64]. Lipases have been applied to adjust the lipid content in fermented black tea, which is one of the most consumed beverages worldwide [65]. Another important sector is the wine industry, where lipases have applications to control the content of acids and esters and thus the aroma [55].

### 3.5. Cosmetic Uses and Personal Care Products

Lipases are used for a variety of purposes in cosmetics and personal care products, for instance, to produce fragrances or bioactive ingredients such as retinol or to adjust the texture and appearance of the final formulation as needed [55]. They have also become widely applied worldwide in various detergents formulations, as they allow for the rapid removal of greasy stains at low temperature, and they offer a green alternative to more aggressive chemical agents [2]. Proteases are also used in detergents formulations [54] and in cosmetic, as whitening agents [66] or to remodel scar tissue [58]. In particular, gel formulations have been developed to maintain the active agents in the desired site of action for prolonged time and to fine-tune their delivery [66,67].

### 3.6. Paper Industry

Various xylanolytic enzymes have been used in the paper industry, individually or in combination, to hydrolyze raw materials. Examples include cellulases, hemicellulases, and xylanases [68]. Lipases are used in the paper industry to promote the rapid removal of ink from waste paper and to increase its whiteness and pulping rate whilst reducing both the energy needed in the industrial process and the need for polluting agents [55].

### 3.7. Leather, Wool, Fur, and Textile Industry

Proteases and lipases are widely used together in blends in the industries that transform animal products into fine leather, wool, and fur. The enzymes not only are helpful for removing the fats, which in some cases are present in high contents, such as in the sheepskins, but also to enhance the quality, stability, appearance, and texture of the desired products. Furthermore, they are used also for similar purposes in the production of textiles such as silk. In the case of cotton, they have another application in processing starch waste into small, water-soluble compounds [55]. In recent years, keratinases have attracted interest for the treatment of industrial waste with high levels of keratin since this biopolymer is generally resistant to protease-mediated degradation, and it can be very persistent in the environment. Relevant sectors include poultry production, which produces a significant amount of feather waste [69], but also others that produce hair and horn byproducts [70,71].

### 3.8. Environmental Remediation

**Figure 5:**
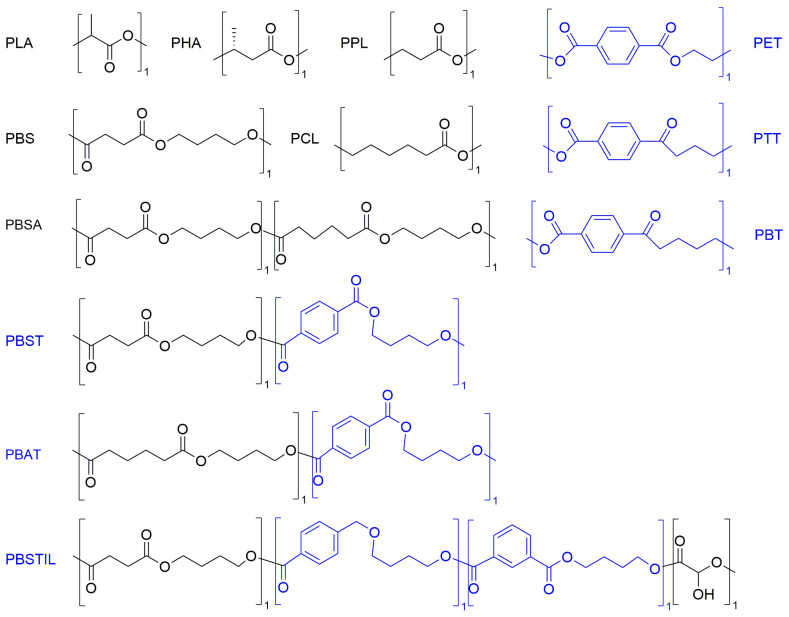
Polymers with aliphatic (black) and aromatic (blue) components that have been tested for hydrolase-mediated biodegradation.

Enzymes have been widely studied for environmental remediation also in gel formulations, for instance, to treat contaminated water [72] and soil [73]. Lipases have been long applied to treat wastewater in several industrial sectors that produce fats, such as the above-mentioned dairy and, more generally, food industries but also in wool and farming activities, for instance, to treat manure. They find use in remediating oil spills, too [55]. More recently, several microbial hydrolases have been applied to the biodegradation of polyester-like plastics (Figure 5). They include depolymerases, esterases, lipases, and cutinases. They perform well on biodegradable polymers, such as poly(L-lactic acid) (PLA), poly(ester carbonate) (PEC), poly(ε-caprolactone) (PCL), poly(propiolactone) (PPL), polyhydroxyalkanoates (PHAs), poly(ethylene succinate) (PES), poly(butylene succinate) (PBS), poly(butylene succinate)-co-(butylene adipate) (PBSA), and also co-polyesters containing aromatic and aliphatic groups, including poly(butylene succinate/terephthalate/isophthalate)-co-(lactate) (PBSTIL) poly(butylene succinate-co-terephthalate) (PBST), and poly(butylene adipate-co-terephthalate) (PBAT). Polymers based solely on aromatic components, however, still pose a challenge, as they are more difficult for hydrolases to biodegrade. These include the widely used poly(ethylene terephthalate) (PET), poly(trimethylene terephthalate) (PTT), and poly(butylene terephthalate) (PBT) [74]. Nevertheless, over the last five years, great progress has been made to find and develop efficient hydrolytic enzymes for the efficient degradation and recycling of these polymers, too [75,76,77].

More recently, dehalogenases have drawn attention for environmental remediation of organohalides, which are notoriously known for their persistence in the environment [78,79,80]. They include chlorinated [81], brominated [82], and fluorinated [83] species, such as pesticides and waste products from various industrial activities. Lastly, keratinases have attracted recent interest for the sustainable management of industrial waste that is composed of keratinous masses, especially derived from animal processing, as mentioned in the above Section 3.7 [69,70,71].

### 3.9. Biosensing

Lipases have been used in biosensors for the quantification of not only triacylglycerols, which are their natural substrates, but also pesticides. The types of sensors include electrochemical as well as optical thanks to the enzyme’s ability to convert chromogenic substrates into colored products [84].

### 3.10. Biodiesel Production

Lipases are robust enzymes with promiscuous activity and good tolerance towards variations in temperature, presence of solvents, and a variety of chemicals. In particular, microbial lipases have been successfully employed for the production of biodiesel thanks to these advantageous features [85].

## 4. Hydrolase Supramolecular Mimicry by Self-Assembling Peptides

Several peptide conjugates have been designed over the years so that they can self-assemble into supramolecular structures for hydrolase biomimicry. An excellent recent review exists on the topic [86]; therefore, in this work, we focus more specifically on peptides as opposed to their derivatives, such as peptide amphiphiles.

**Figure 6:**
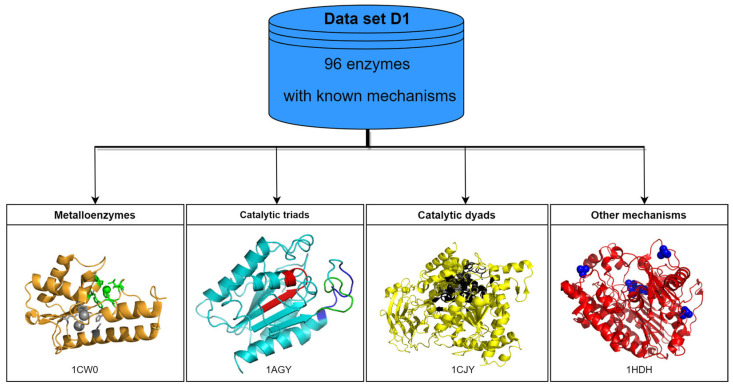
Schematic representation of the 96 esterase enzymes (EC 3.1) from the M-CSA (EMBL-EBI) database grouped according to their mechanism of action. Representative examples of each mechanism are shown: VSR endonuclease (PDB ID: 1CW0) for metalloenzymes, cutinase (PDB ID: 1AGY) for triads, cytosolic phospholipases A2 (PDB ID: 1CJY) for dyads, and arylsulfatase (PDB ID: 1HDH) for other mechanisms. Adapted with permission from [23], Copyright © 2022, American Chemical Society.

Hydrolases are a heterogenous group of hydrolytic enzymes with diverse mechanisms used to exert catalytic activity. However, the vast majority feature one histidine (His) amino acid in the catalytic site to promote proton abstraction from water to produce the nucleophilic hydroxy-anion that can attack the substrate to hydrolyze a chemical bond (e.g., an ester, an amide, and so on, as described in Section 2 above) [87]. This His can be activated in various ways, for instance, by metal coordination in metalloproteins or by other residues constituting catalytic dyads or triads, as shown in Figure 6 for esterases as the representative example [23].

Numerous efforts have been devoted to produce self-assembling peptides with His residues to attain supramolecular architectures that could mimic hydrolases, and detailed descriptions of earlier works in the field can be found in older [88] and more recent [87] research. Typical designs feature β-structured amphiphilic sequences with alternating hydrophobic and hydrophilic amino acids so as to attain β-sheets with one hydrophobic surface that promotes self-assembly in water and one hydrophilic surface that mediates catalysis. This type of design is apparent also from the sequences reported in Table 1, which summarizes the newest supramolecular peptide hydrolytic biocatalysts produced in the last 5 years.

A common feature of the peptide sequences reported in Table 1, besides their amphiphilic nature, is the presence of Phe. This aromatic residue typically is not meant to exert a catalytic role but rather a structural role thanks to the unmatched fibrillating ability that it displays alone [97] or even better when present in multiple residues or repeats [98,99,100,101,102,103].

In recent years, several attempts have been made to include amino acid residues from catalytic triads in short peptide sequences able to self-assemble into discrete nanostructures or nanostructured hydrogels. However, it is not yet clear how to position them effectively to reproduce the three-dimensional catalytic surface of enzyme active sites. A recent work led by Rapaport examined three amphiphilic peptides with β-sheet structures, each containing a different arrangement of the catalytic triad amino acids Glu, His, and Ser along the strands. Their ability to efficiently hydrolyze the substrate *p*-nitrophenyl acetate (pNPA) was assessed under mild conditions (pH 7, 25 °C), and it was demonstrated that Ac-CFEFSFHFP-NH_2_ possessed the highest catalytic efficiency, achieving a value of 0.19 M^−1^ s^−1^ at a peptide concentration of 0.25 mM at pH 7, in which the peptide was assembled into elongated aggregates [89].

The spatial positioning of Ser near His as well as the right level of dynamicity in the peptide assemblies (intended as a balance between their flexibility and rigidity) appeared to be key factors for achieving the highest catalytic efficiency, which is in agreement with previous studies [104]. However, the mere adjacent positioning of Ser and His in self-assembling short peptides is not enough to improve catalytic activity. Actually, examples were recently reported showing that the addition of Ser adjacent to His in an established supramolecular biocatalytic hydrogel had a detrimental effect on the catalytic performance for ester hydrolysis [105].

Besides Ser, Cys is another nucleophilic amino acid that has been used, in addition to His, to attain better catalytic performances. A recent study by Kleinsmann and Nachtsheim demonstrated that Cys and His cooperatively exert the catalytic activity in minimalistic cyclodipeptide co-assembled gels, whereby introduction of Phe was used to promote fibrillation in water and to stabilize the resulting hydrogels through CH–π interactions [91].

The effect of peptide termini on the catalytic performance was also studied. Interestingly, in the case of the minimalistic peptide sequence hFF (where h indicates D-His), it was found that C-amidation [106] enhanced the catalytic performance and the hydrogel stability relatively to the free carboxylic acid terminus [107], demonstrating that the latter one did not mimic the acidic residue that is often present in the catalytic active sites [106]. Conversely, N-acetylation impeded correct self-assembly into hydrogels and slightly negatively affected the catalytic performance [106]. However, it is worth noting that the free N-terminus could also undergo undesired acylation by acting as a nucleophile [90].

**Figure 7:**
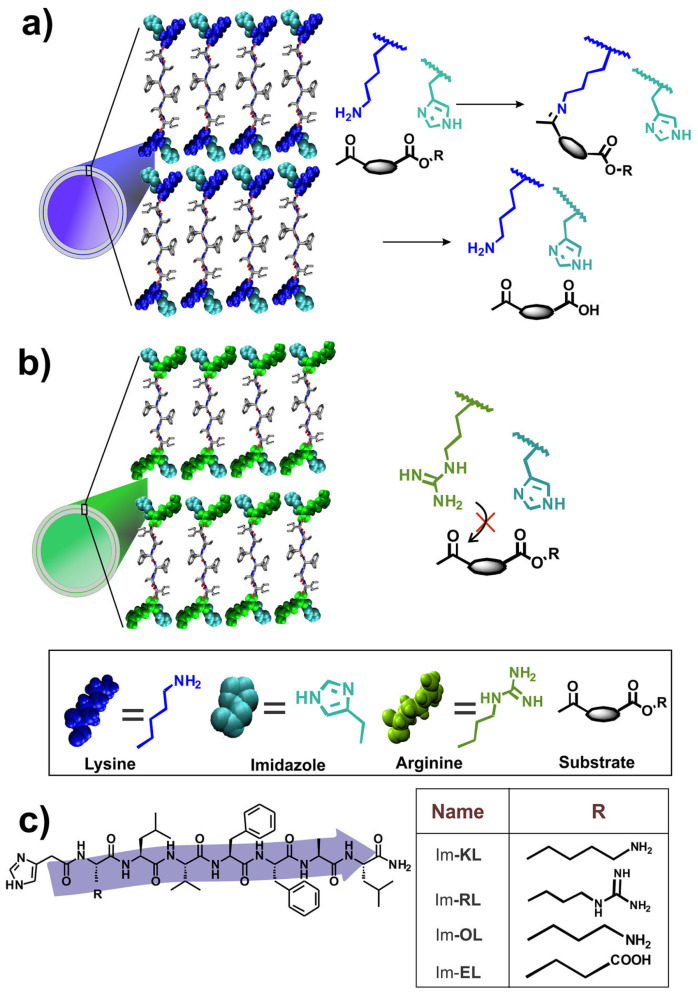
Schematic representation of biocatalytic peptide nanotubes acting via covalent activation through imine formation. (**a**) Blue nanotubes with Lys, (**b**) green nanotubes with Arg, and (**c**) peptide sequences variations for the biocatalysts. Reproduced with permission from [92], Copyright © 2020, American Chemical Society.

Interestingly, a recent work demonstrated that the nucleophilic reactivity of primary amines present in peptide sequences can actually be used in advantageous ways to perform catalysis. In particular, covalent catalysis was demonstrated via imine formation to promote ester hydrolysis and for inactivated esters, too [92]. Four peptide sequences (shown in Figure 7) were designed using the self-assembling LVFFA motif from Alzheimer’s Aβ peptide [108] that was then further elongated with an amidated Leu at the C-terminus and with different residues at the N-terminus that were N-capped with imidazolacetic acid, mimicking His [92].

The same amyloid Aβ motif was used to design other biocatalysts, too. The use of different N-caps based on either the classical fluorenylmethyloxycarbonyl (Fmoc) or 4-methylcoumarin (Cou) led to self-assembling Fmoc-VFFAHH and Cou-VFFAHH, which demonstrated the ability to self-assemble into diverse nanomorphologies that could be interconverted between each other with various stimuli, such as heat and light. In this case, the N-caps served as stimuli-responsive components, while the two His residues at the C-terminus played a key role in the catalysis [93].

A different approach was recently taken by Zhu et al., who molecularly imprinted a polymer hydrogel containing Fmoc-FFH peptide fibers using either one of three ester substrates as templates. They demonstrated that this approach led to enhanced catalytic performance thanks to facilitated substrate binding by the molecularly imprinted polymer [94].

More recently, phosphatase mimicry was also reported by self-assembling short peptides. In this case, helical heptapeptides were designed featuring different positioning of Ser and His [95] within amphiphilic sequences that contained α-aminoisobutyric acid for promoting helical propensity thanks to more limited conformational freedom [109]. The helices assembled into coils that yielded nanofibrils in both cases. The heptapeptide with the Ser at the N-terminal position demonstrated higher catalytic efficiency than the analog with the His at the N-terminus. The authors used in silico studies to demonstrate that the enhanced activity of the former was due to the presence of the Ser nucleophile in proximity of the N-terminus, which electrostatically bound to the negatively charged phosphate group of the substrate [95].

Overall, these studies advanced our understanding of the various factors that come into play when designing supramolecular biocatalysts for hydrolase mimicry. Yet, a boost in catalytic efficiency can simply be attained by His activation through metal coordination to divalent Zn(II). The latest advancement in this area showed that His is not even required in this case, and a biocatalyst as simple as Phe coordinated with Zn(II) can reach a catalytic efficiency as high as 76.5 M^−1^ s^−1^ [96]. All things considered, metal coordination can be advantageous not only to boost catalytic activity but also the rheological properties of the resulting gels [110,111,112,113].

## 5. Multifunctional Supramolecular Biocatalysts Based on Hydrolytic Peptides

The research area of enzyme mimicry by means of self-assembling peptides is finally reaching maturity, as it is moving towards biochemical cascades and multifunctional biocatalysts. This concept has already been successfully applied to enzymes through various approaches [114], such as genetic fusion [115]. Also, in this case, nature provides a great source of inspiration [116,117]. For instance, carbonic anhydrase is an enzyme capable of dual activity, consisting of both the hydration of carbon dioxide and ester hydrolysis [118]. Typically, the active site displays three His residues coordinating Zn(II). This feature inspired an elegant study led by Gazit, whereby a minimalistic biocatalyst was attained from the amino acid Phe coordinating Zn(II). Interestingly, the crystals displayed catalytic activity mimicking carbonic anhydrase, exceeding the performance of the natural enzyme in ester hydrolysis considering the low molecular mass of the supramolecular biocatalyst. Remarkably, the asymmetric packing in the crystal enabled the stereoselective hydrolysis of chiral esters, such as those of N-protected amino acids (i.e., Boc-Phe), with the chromogenic *p*-nitrophenol unit [96], which enabled the rapid screening of catalytic activity by a simple colorimetric assay as long as the pH was controlled to avoid acid-catalyzed or base-catalyzed hydrolysis [119].

**Figure 8:**
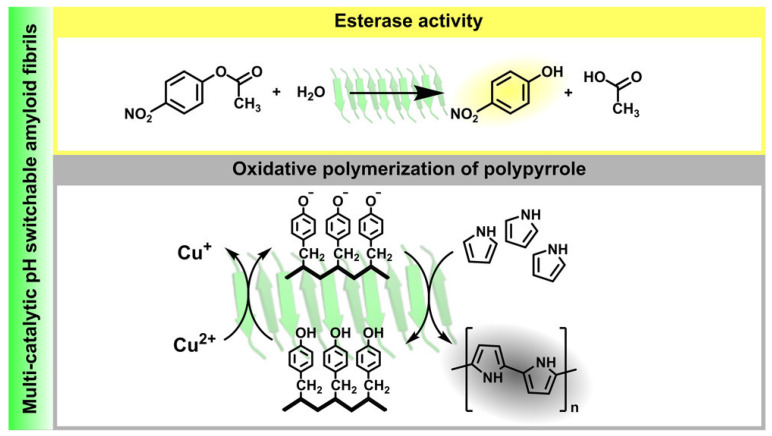
Schematic representation of dual enzymatic activity of Tyr-His supramolecular amyloids: esterase-like activity (yellow, top) and oxidative polymerization of pyrrole (grey, bottom). Reproduced with permission from [120], Copyright © 2020, American Chemical Society.

A research work led by Ventura showed that simple Tyr-His repeats could self-assemble into biocompatible amyloid fibrils that demonstrated dual enzymatic activity (Figure 8). The first one was based on ester hydrolysis and the latter on the oxidative polymerization of pyrrole in the presence of Cu(II) ions [120].

Surprisingly, even natural amyloids can display dual catalytic activity. Jelinek and colleagues demonstrated that the Alzheimer’s-disease-associated amyloid β peptide Aβ42 fibrils could exert both hydrolytic and oxidative catalysis. In the former case, the anisotropic nanostructures catalyzed the breakdown of not only model substrates such as *p*-nitrophenyl acetate but also of acetylthiocholine as a model of the neurotransmitter acetylcholine. In the latter case, the full-length amyloids could promote the oxidation of neurotransmitters dopamine and adrenaline, with lower catalytic activity performed by shorter fragments of the naturally occurring peptide. Furthermore, the most active biocatalysts were the mature fibrils as opposed to Aβ monomers or soluble oligomers [121]. This study suggested that the fibrils occurring in Alzheimer’s disease may play some insidious catalytic role in the degradation of neurotransmitters during the pathological process of neurodegeneration.

The possibility to engineer both hydrolytic and oxidative catalysis sequentially in a cascade was explored in a recent study led by Das. In this work, nanotubes were formed by the self-assembly of Ac-HLVFFAL-NH_2_. The nanostructured surface exposed catalytically active His residues for hydrolase mimicry that were able to bind hemin, too, for peroxidase mimicry. The dual and sequential activity was demonstrated with 2-methoxy phenyl acetate (MPA) as substrate, which was converted to tetraguaiacol as final product [122].

## 6. Conclusions

Enzyme mimicry through supramolecular peptide assembly is a research area that is reaching maturity. Considering the multifarious applications of hydrolytic enzymes in several industrial sectors, it is not surprising to note how hydrolase mimicry is the most studied type of catalytic activity displayed by self-assembled peptides. Furthermore, the newest developments in the field are combining this type of reactivity with other modes of catalysis to attain sequential transformations in cascade reactions in a similar manner as tandem enzymatic catalysis occurs in natural biochemical pathways.

In general, design rules are emerging to attain catalytically active peptide assemblies. However, there are still many unanswered questions, such as how to effectively reproduce the three-dimensional spatial positioning of catalytic residues within a supramolecular architecture, as featured in natural enzymes. Indeed, in this concise review, we have discussed examples whereby simple designs (e.g., positioning of Ser adjacent to His) can enhance catalytic activity in some cases and worsen it in others for reasons that are not always crystal clear.

Nowadays, our modern society faces the urgency of solving the most compelling global challenges, such as hunger, climate change, resource depletion, and environmental remediation. Enzymes have traditionally played an elected role in developing sustainable production and remediation methods, and over the years, several engineering studies have produced numerous variants with improved stability and activity. Nevertheless, enzymes also present some limitations and drawbacks, such as their high molecular weight, allergenicity, and susceptibility towards degradation upon prolonged use.

Therefore, it is evident that minimalistic self-assembling peptide gels are attractive alternatives to circumvent these issues. Areas of particular relevance for future developments pertain to applications such as medicine, efficient biodiesel production, microplastics and nanoplastics degradation, and, more generally, environmental remediation from persistent pollutants. Gels show high potential to innovate in these areas, and their combination with nanotechnology is particularly promising [123,124,125,126]. Furthermore, many industrial sectors could benefit from embracing the use of enzymes in gels for various purposes, with clear advantages being presented by the most modern systems with sequential enzymatic activity. Finally, the creative combination of catalytic units with self-assembling motifs offers a promising approach to develop sustainable biocatalysts. Recent examples include the use of photoactive units [127,128,129,130]. In particular, the use of photo-responsive moieties has been applied to organic transformations [127,128], such as cis-trans alkene photoisomerization [127] and photo-oxidation of organic sulfides to sulfoxides [128] and to hydrogen production [129,130]. We can anticipate that in the future, photo-organocatalysis coupled with the chiral nature of peptides to attain stereoselectivity could open up very interesting avenues to render further industrial processes more sustainable for the benefit of the whole society.

## Figures and Tables

**Table 1:** Supramolecular peptide biocatalysts for esterase mimicry reported since 2019. Underlined amino acids are those that play key roles in hydrolase catalytic active sites.

Peptide Sequence	Assembly Type	Reaction Conditions	Substrate	Catalytic Efficiency ^1^	Refs.
Ac-CFEFSFHFP-NH_2_	Aggregates Fibrils	pH 7, 25 °C	p-NPA ^2^	ε = 0.19 M^−1^ s^−1^	[89]
Ac-CFHFEFSFP-NH_2_	Aggregates Fibrils	pH 7, 25 °C	p-NPA ^2^	ε = 7.59 · 10^−2^ M^−1^ s^−1^	[89]
Ac-CFEFHFSFP-NH_2_	Aggregates Fibrils	pH 7, 25 °C	p-NPA ^2^	ε = 4.08 · 10^−2^ M^−1^ s^−1^	[89]
HLlIHLlI	Fibrils Hydrogel	pH 7, 25 °C	p-NPA ^2^	ε = 2.9 M^−1^ s^−1^	[90]
Cyclo(HF) + Cyclo(CL)	Fibrils Hydrogel	pH 7, 25 °C	p-NPA ^2^	ε = 0.2 M^−1^ s^−1^	[91]
Cyclo(HF) + Cyclo(CF)	Fibrils Hydrogel	pH 7, 25 °C	p-NPA ^2^	ε = 0.09 M^−1^ s^−1^	[91]
Im-KLVFFAL-NH_2_ ^3^	Nanotubes	pH 7, 25 °C	p-NPOP ^4^	ε = 2.1 M^−1^ s^−1^	[92]
Im-KLVFFAL-NH_2_ ^3^	Nanotubes	pH 7, 25 °C	p-NPP ^5^	ε = 3.6 M^−1^ s^−1^	[92]
Im-RLVFFAL-NH_2_ ^3^	Nanotubes	pH 7, 25 °C	p-NPOP ^4^	ε = 0.5 M^−1^ s^−1^	[92]
Im-RLVFFAL-NH_2_ ^3^	Nanotubes	pH 7, 25 °C	p-NPP ^5^	ε = 1.8 M^−1^ s^−1^	[92]
Fmoc-VFFAHH	Nanofibers	pH 7, 25 °C	p-NPA ^2^	ε = 1.0 M^−1^ s^−1^	[93]
Fmoc-VFFAHH	Twisted bundles	pH 7, 25 °C	p-NPA ^2^	ε = 1.71 M^−1^ s^−1^	[93]
Cou-VFFAHH ^6^	Nanofibers	pH 7, 25 °C	p-NPA ^2^	ε = 0.575 M^−1^ s^−1^	[93]
Fmoc-FFH	MIP ^7^	pH 8, 25 °C	p-NPA ^2^	ε = 15.5· 10^−3^ mM^−1^ min^−1^	[94]
Fmoc-FFH	MIP ^7^	pH 8, 25 °C	p-NPB ^8^	ε = 11.2· 10^−3^ mM^−1^ min^−1^	[94]
Fmoc-FFH	MIP ^7^	pH 8, 25 °C	p-NPH ^9^	ε = 5.65· 10^−3^ mM^−1^ min^−1^	[94]
S*A*FH*A*F*A* ^10^	Fibrils	pH 7, 37 °C	p-NPA ^2^	ε = 1.07 M^−1^ s^−1^	[95]
H*A*FS*A*F*A* ^10^	Fibrils	pH 7, 37 °C	p-NPA ^2^	ε = 0.90 M^−1^ s^−1^	[95]
S*A*FH*A*F*A* ^10^	Fibrils	pH 7, 37 °C	p-NPp ^11^	ε = 0.70 M^−1^ s^−1^	[95]
H*A*FS*A*F*A* ^10^	Fibrils	pH 7, 37 °C	p-NPp ^11^	ε = 0.28 M^−1^ s^−1^	[95]
F-Zn(II)	Crystals	pH 7, 25 °C	p-NPA ^2^	ε = 76.5 M^−1^ s^−1^	[96]

^1^ Parameters are reported for the best performance. ^2^ pNPA, *p*-nitrophenyl acetate; ^3^ Im, imidazoleacetyl; ^4^ pNPOP, *p*-nitrophenyl 4-oxopentanoate; ^5^ pNPP, *p*-nitrophenyl pentanoate; ^6^ Cou, 4-methylcoumarin; ^7^ MIP, substrate molecular imprinted polymeric hydrogel with peptide fibrils; ^8^ pNPB, *p*-nitrophenyl butyrate; ^9^ pNPH, *p*-nitrophenyl hexanoate; ^10^ *A*, α-aminoisobutyric acid; ^11^ pNPp, *p*-nitrophenylphosphate.

## Data Availability

Not applicable.
